# Supplementary material for: Comparison between Ultrasonographic-Guided Temporal and Coronoid Approaches for Trigeminal Nerve Block in Dogs: A Cadaveric Study
Source: Animals (Basel). 2024 May 31;14(11):1643. doi: 10.3390/ani14111643 (PMC11171064; doi:10.3390/ani14111643)

SUPPLEMENTARY MATERIAL

Supplementary Figure S1. Anatomical dissection of the Pterygopalatine fossa.

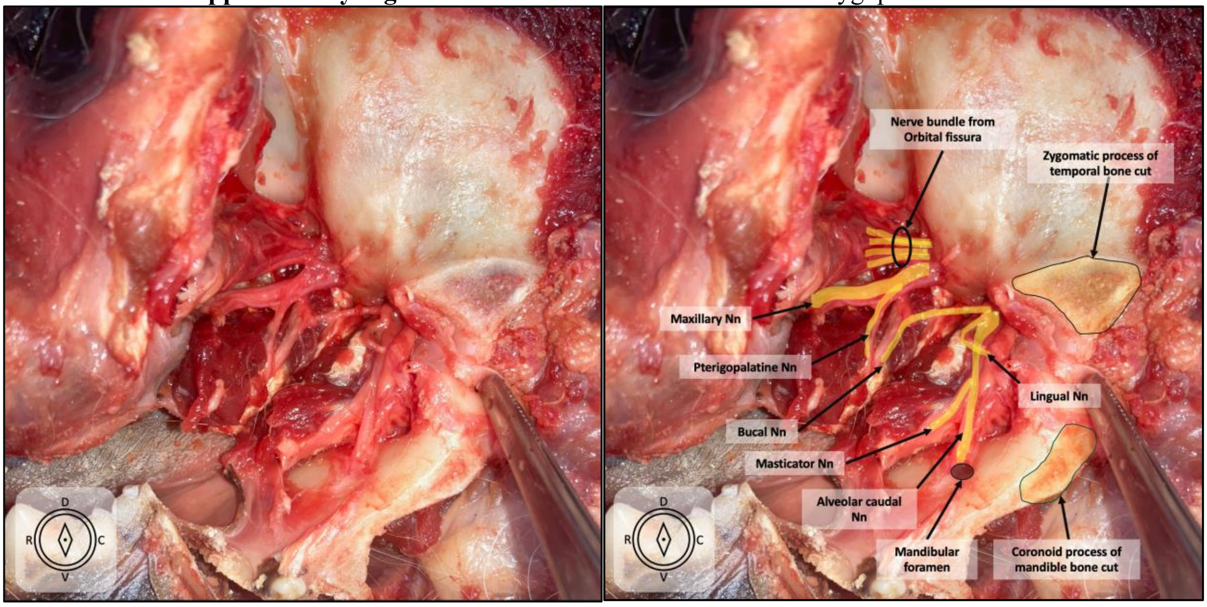

Supplementary Figure S2. Craneal length (CrL)

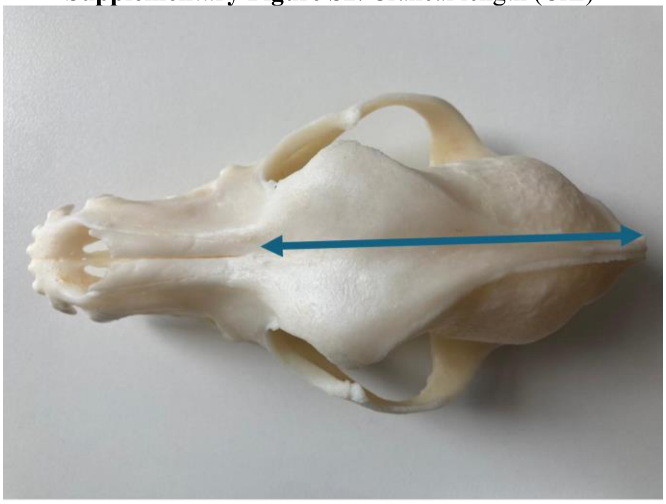

Supplementary Figure S3. Transverse CT image displayed in bone window and mildly angulated to the needle plane, with the needle in the coronoid approach (Left side of the screen). Note the position of the tip of the needle in the proximity of the orbital fissure and rostral alar foramen

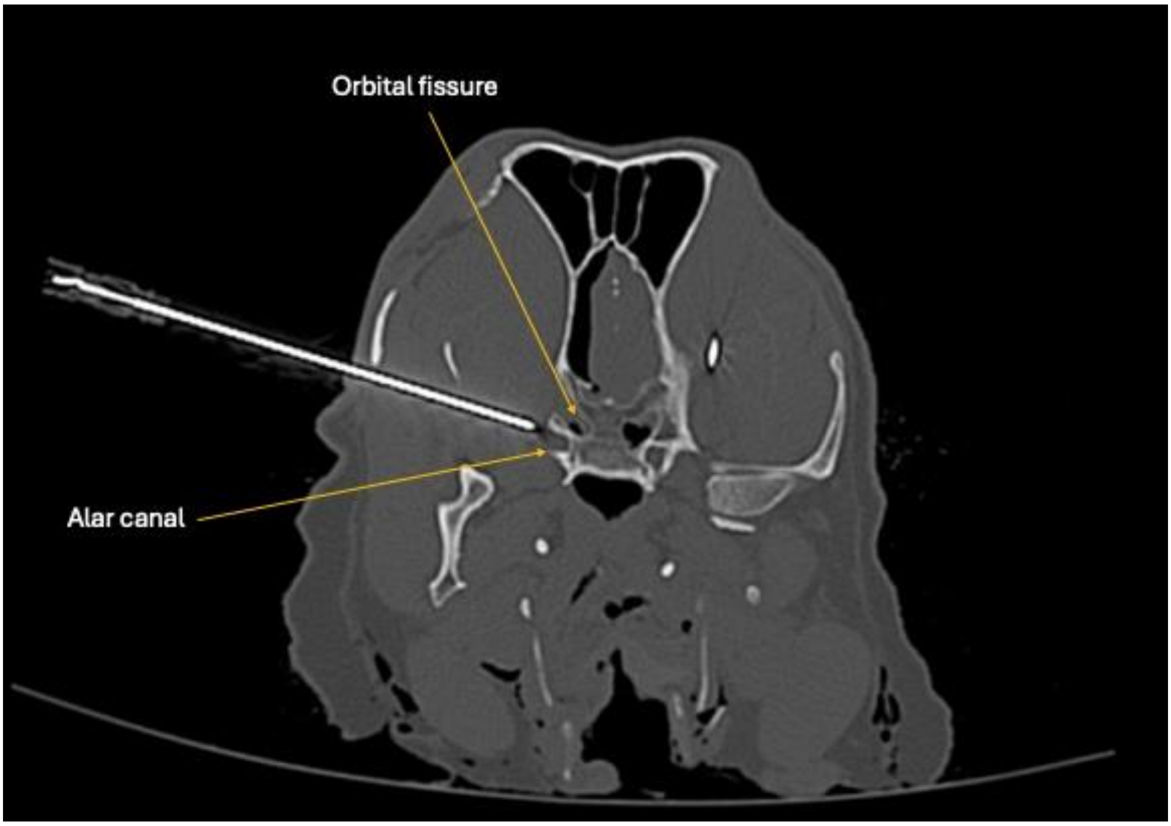

Supplementary figure S4. Transverse CT image displayed in bone window and mildly angulated to the needle plane at the level of the orbital fissure, with the needle in place in the temporal approach (right side of the screen)

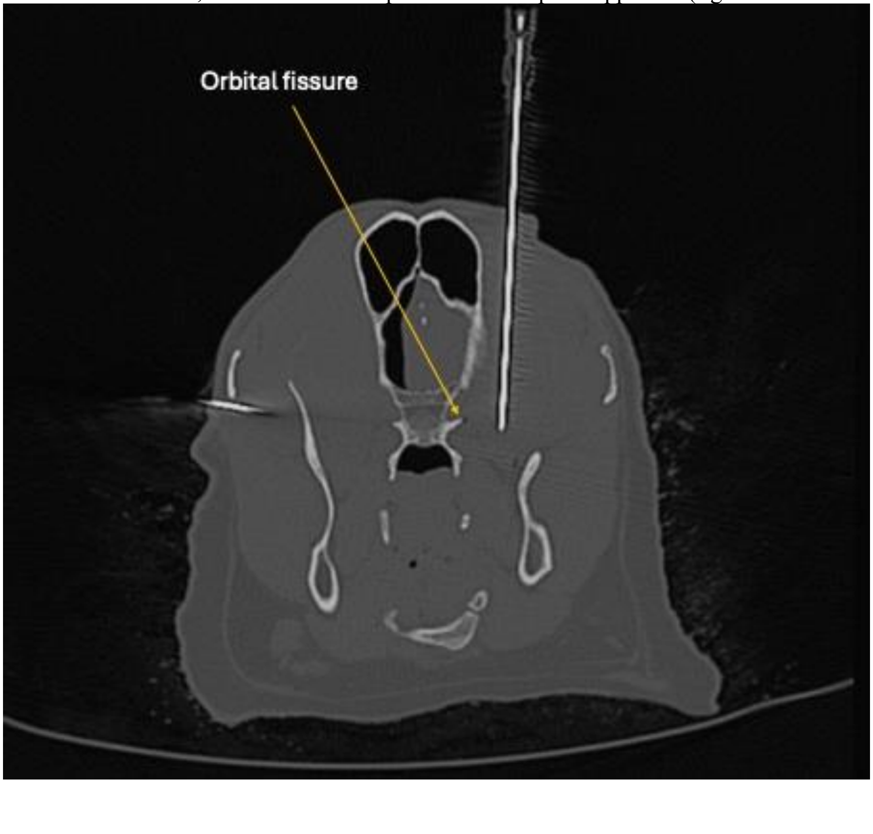

Supplementary Figure S5. Transverse CT image of the head of a dog at the level of the alar canal and orbital fissure, displayed in bone window, where contrast medium can be observed over the surface of the frontal and sphenoid bones, and inside the orbital fissure after an injection using a coronoid approach (left side of the screen).

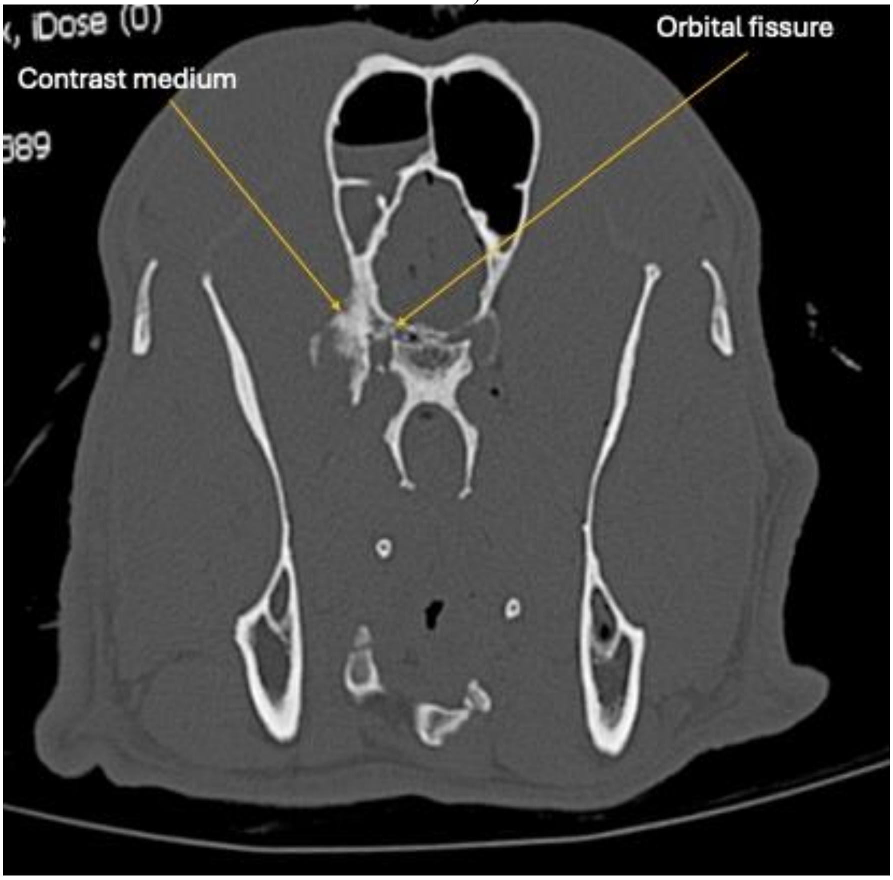

Supplementary Figure S6. 3D reconstruction of the head of a dog after placement of the needle with temporal approach.

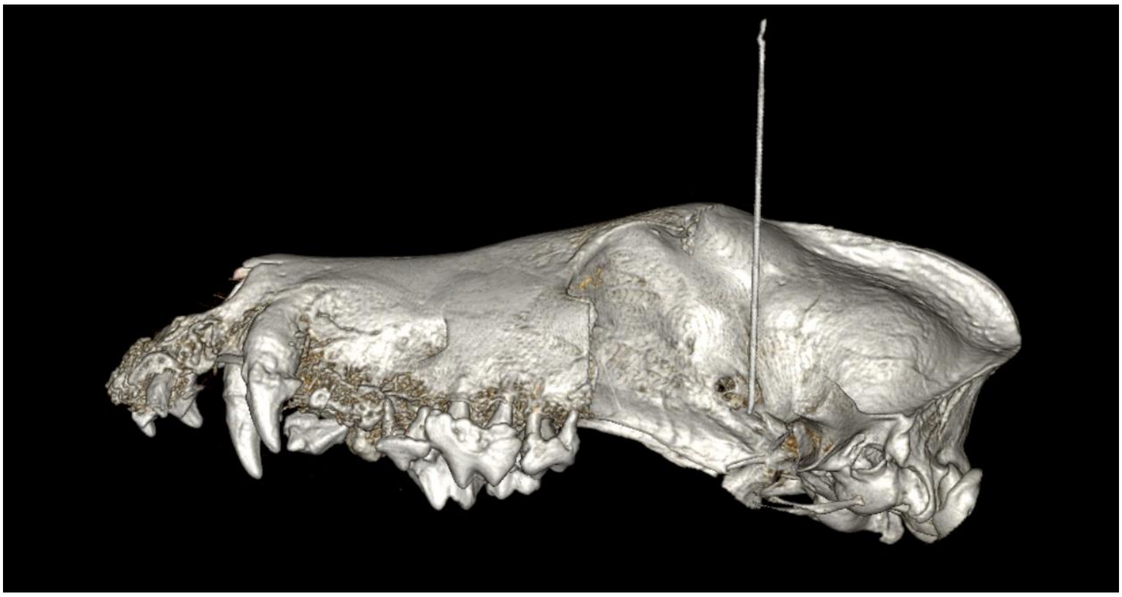

.

Supplementary figure S7. 3D reconstruction of the head of a dog after placement of the needle with coronoid approach.

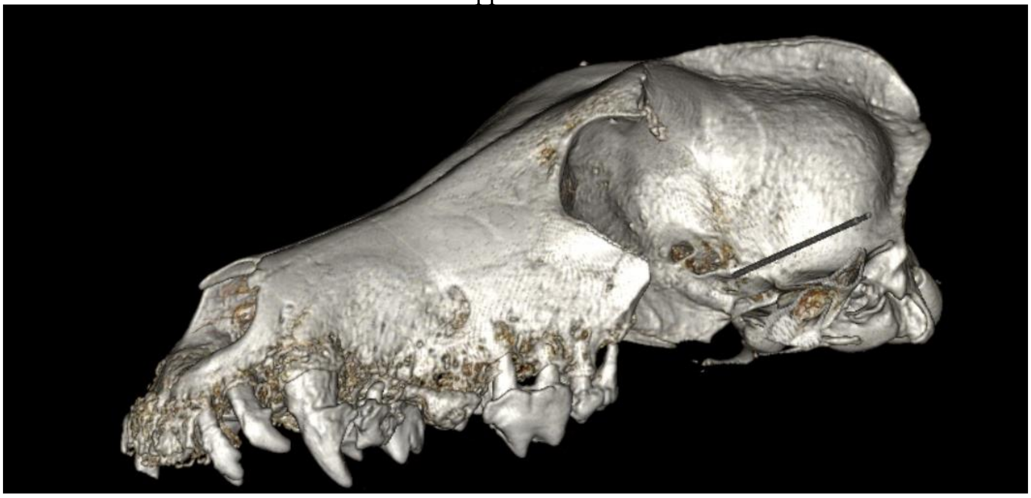

Figure S8. CT study in ten specimens. Blue colour indicates presence of the contrast within the structure of interest (cranium, retrobulbar conus, muscle, interfascial) or in contact with it (optic canal, orbital fissure, rostral alar canal, foramen ovale). Red colour indicates absence within or non contact with it..

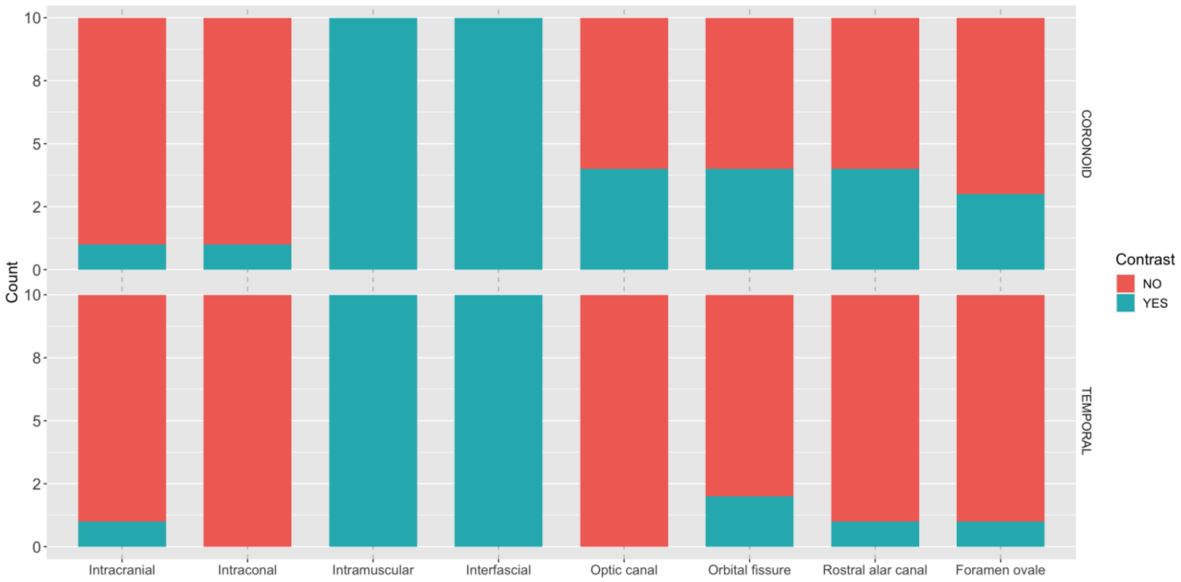

Figure S9. Dissection study in ten specimens. Blue colour indicates presence of dye within the structure of interest (retrobulbar conus, muscle, interfascial) or staining of the nerve (mandibular, maxillary, optic nerves and orbital fissure complex).

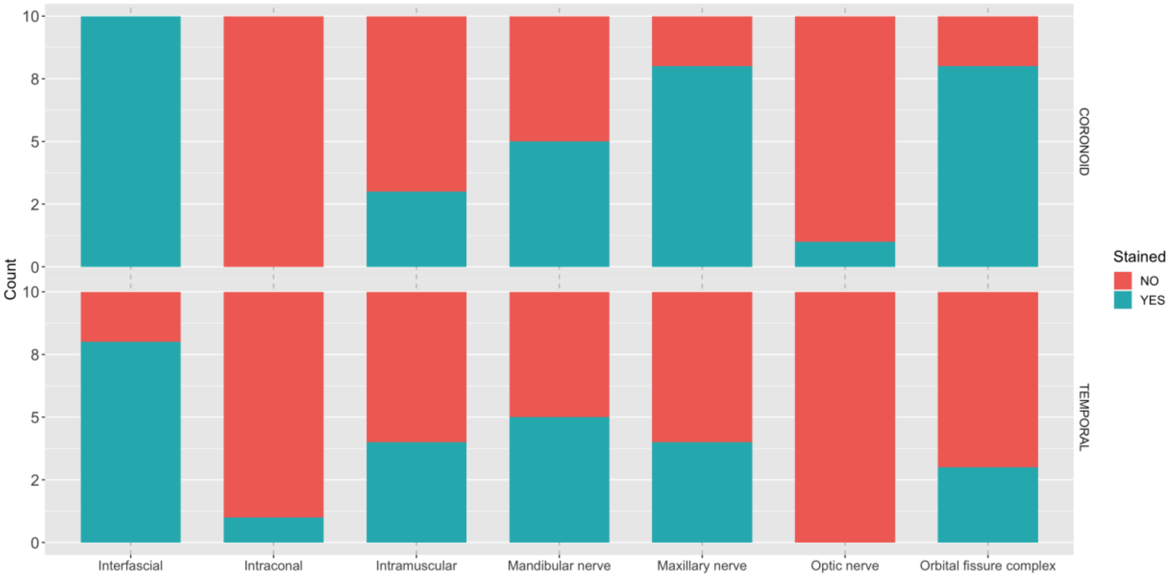

Supplement: Supplementary file 1 [file animals-14-01643-s001.zip › animals-3025851-supplementary.pdf]
